# Supplementary material for: TM9SF2 Maintains Golgi Integrity and Regulates Ricin-Induced Cytotoxicity
Source: Toxins (Basel). 2025 Apr 26;17(5):218. doi: 10.3390/toxins17050218 (PMC12116000; doi:10.3390/toxins17050218)
Supplement: Supplementary file 1 [file toxins-17-00218-s001.zip › toxins-3556867-supplementary 1.pdf]

Supplementary Materials: TM9SF2 Maintains Golgi Integrity and Regulates Ricin-Induced Cytotoxicity

Yue Meng, Hongzhi Wan, Xinyu Wang, Lina Zhang, Ruozheng Xin, Lingyu Li, Yuhui Wang, Chengwang Xu, Hui Peng, Lu Sun, Bo Wang and Xiaotao Duan

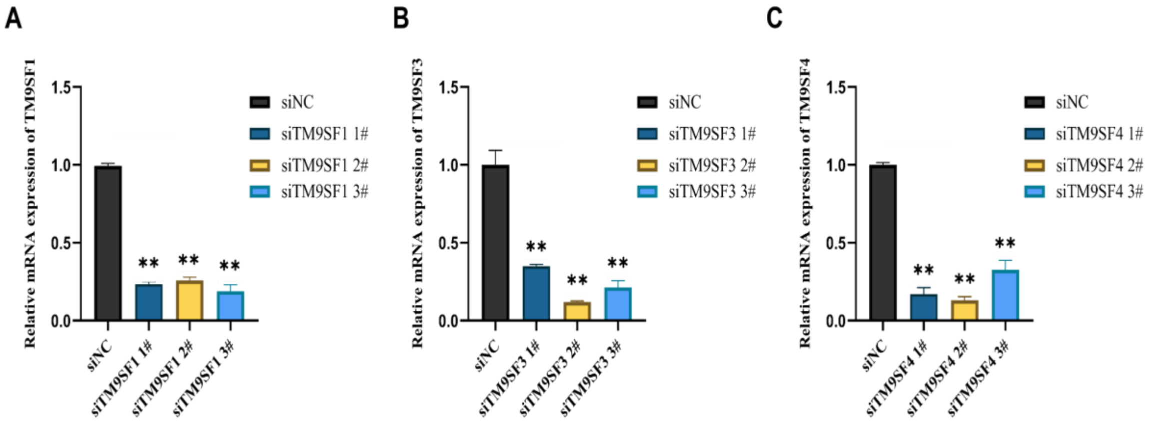

Figure S1. Generation of TM9SF family member knockdown cell populations using siRNA technology, Related to Figure 1. C-E) Relative quantities of the mRNA transcript levels of TM9SF1 (A), TM9SF3 (B), and TM9SF4(C) observed in the different group cells (** $p < 0.01$, vs. siNC group, n = 3).

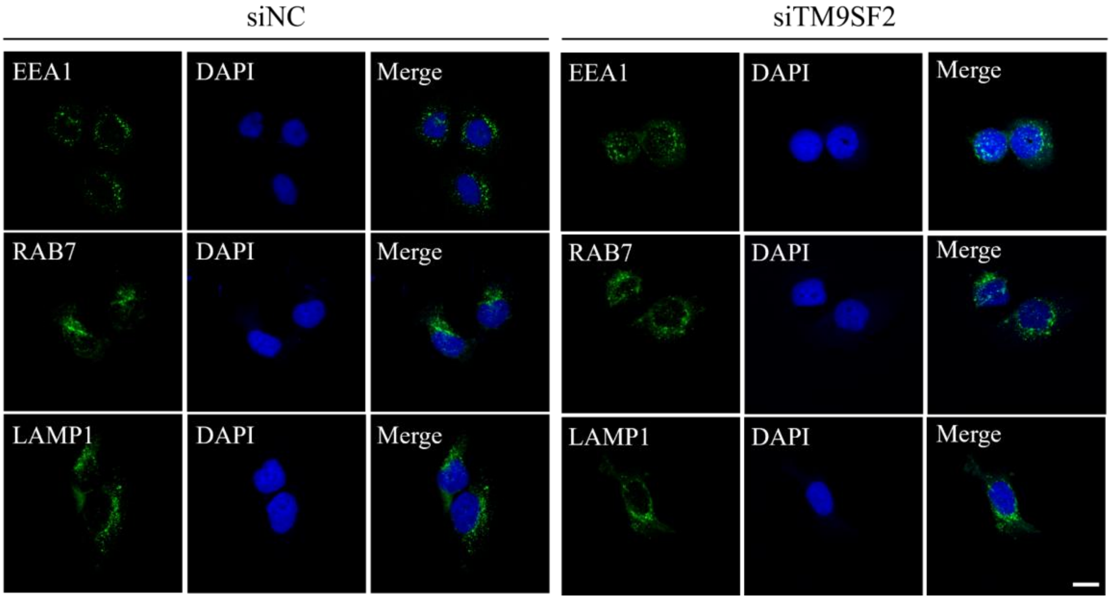

Figure S2. TM9SF2 silencing does not affect the morphology of early endosomes, late endosomes, and lysosomes. Related to Figure 3. A) At 36 h post-transfection, siNC or silenced cells were fixed and stained for different organelle markers, EEA1 represents early endosomes, RAB7 represents late endosomes, LAMP1 represents lysosomes. Scale bar, 10 μm.

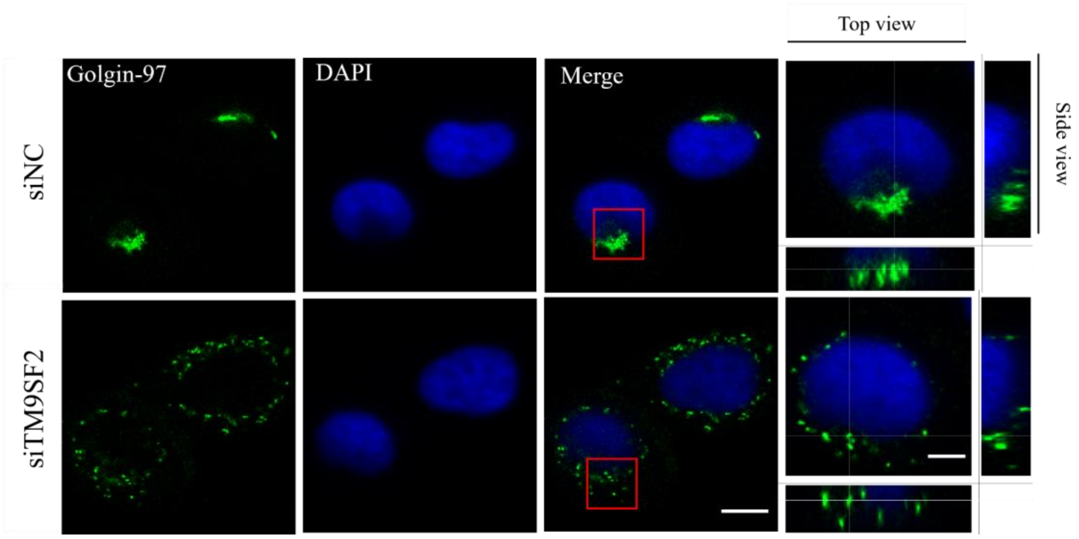

Figure S3. TM9SF2 knockdown affects the morphology of the Golgi apparatus. Related to Figure 3. A) At 36 h post-transfection, siNC or silenced cells were fixed and stained for Golgin-97 (in green) and DAPI (in red) examined by means of confocal microscopy (z- scan). Scale bar, 10 µm, 5 µm.
